# Chromatin Modification by PSC Occurs at One PSC per Nucleosome and Does Not Require the Acidic Patch of Histone H2A

**DOI:** 10.1371/journal.pone.0047162

**Published:** 2012-10-11

**Authors:** Stanley M. Lo, Kyle A. McElroy, Nicole J. Francis

**Affiliations:** Department of Molecular and Cellular Biology, Harvard University, Cambridge, Massachusetts, United States of America; National Cancer Institute, United States of America

## Abstract

Chromatin architecture is regulated through both enzymatic and non-enzymatic activities. For example, the Polycomb Group (PcG) proteins maintain developmental gene silencing using an array of chromatin-based mechanisms. The essential *Drosophila* PcG protein, Posterior Sex Combs (PSC), compacts chromatin and inhibits chromatin remodeling and transcription through a non-enzymatic mechanism involving nucleosome bridging. Nucleosome bridging is achieved through a combination of nucleosome binding and self-interaction. Precisely how PSC interacts with chromatin to bridge nucleosomes is not known and is the subject of this work. We determine the stoichiometry of PSC-chromatin interactions in compact chromatin (in which nucleosomes are bridged) using Scanning Transmission Electron Microscopy (STEM). We find that full compaction occurs with one PSC per nucleosome. In addition to compacting chromatin, we show that PSC oligomerizes nucleosome arrays. PSC-mediated oligomerization of chromatin occurs at similar stoichiometry as compaction suggesting it may also involve nucleosome bridging. Interactions between the tail of histone H4 and the acidic patch of histone H2A are important for chromatin folding and oligomerization, and several chromatin proteins bind the histone H2A acidic patch. However, mutation of the acidic patch of histone H2A does not affect PSC’s ability to inhibit chromatin remodeling or bridge nucleosomes. In fact, PSC does not require nucleosomes for bridging activity but can bridge naked DNA segments. PSC clusters nucleosomes on sparsely assembled templates, suggesting it interacts preferentially with nucleosomes over bare DNA. This may be due to the ability of PSC to bind free histones. Our data are consistent with a model in which each PSC binds a nucleosome and at least one other PSC to directly bridge nucleosomes and compact chromatin, but also suggest that naked DNA can be included in compacted structures. We discuss how our data highlight the diversity of mechanisms used to modify chromatin architecture.

## Introduction

Eukaryotic DNA is packaged into nucleosomes and further folded and looped into higher order chromatin structures. Regulatory elements which can interact over long distances and even in *trans* contribute to these structures. The structures of folded chromatin, the role of non-histone proteins in forming and regulating them, and their direct functional consequences have been difficult to elucidate. Several proteins that can compact nucleosomal arrays and oligomerize them into large structures have been described [1]–[4]. These proteins differ in the domains they use to interact with chromatin, their stoichiometry of binding to chromatin, and the mechanism by which they promote changes in chromatin structure. This suggests different proteins form specific types of chromatin architectures that serve regulatory functions. One example of chromatin architecture proteins are the Polycomb Group (PcG) proteins.

PcG genes were initially identified in *Drosophila* [5], [6] and are conserved throughout metazoans and in plants [7], [8]. They play key roles in regulating *Hox* gene expression during development [9] and in other processes such as mammalian X-inactivation [10], genomic imprinting [11], cell cycle progression, and differentiation and self-renewal of various mammalian and *Drosophila* stem cells [12]–[14]. PcG proteins are thought to maintain gene silencing through covalent modification of histone proteins [12] and non-covalent modification of chromatin [15], [16] and DNA [17] architecture.

Evidence from both *Drosophila* and mammals implicates PcG proteins in chromatin compaction and long range and *trans* chromatin interactions. In *Drosophila*, Polycomb Response Elements (PREs), which bind PcG proteins and silence target genes, can function over long distances [18]. Second, many PREs mediate pairing sensitive silencing (PSS), in which homozygous transgenes are silenced more effectively than heterozygous ones; mutations in PcG genes disrupt PSS (reviewed in [19]). Third, transposons containing PREs tend to integrate near other PcG binding sites in the genome [20], [19]. PREs involved in gene silencing have been observed in close physical proximity by FISH [21], [22] but see [23]–[24]. Chromosome Conformation Capture (3C) was used to identify extensive long range interactions among multiple PREs in the Bithorax complex (BX-C) and among PREs and repressed gene promoters and 3′ ends [22]. These interactions were decreased after reduction of PcG protein expression. More recently, high throughput analyses have identified extensive domains of long-range interactions by PcG bound sites [25]–[27]. Recent data implicate insulator proteins in forming long range interactions between PREs in *Drosophila* suggesting that *trans* interactions among PcG protein bound sites may occur after these regions are brought together through the activities of insulator proteins [28], [29]. PcG proteins are also associated with chromatin compaction in mouse. In mouse embryos, two loci which bind PcG proteins, *Kcnq1* [30] and *HoxB* [31], are compacted. *HoxB* and another *Hox* cluster, *HoxD*, are also compacted in mouse embryonic stem cells (mES) and decompacted upon differentiation induced gene activation [32], [33]. Loss of PcG proteins leads to decompaction of the *Kcnq1* locus in mouse embryos [30] and of the *HoxB* and *HoxD* clusters in mES cells [33]. Thus, extensive evidence links PcG proteins to long range interactions and changes to chromatin conformation. The mechanistic basis of chromatin compaction observed *in vivo* is not clear since local compaction of chromatin through either alterations to chromatin folding or formation of chromatin loops can shorten the distance between distal sequences, which is the assay typically used to demonstrate compaction *in vivo*. Furthermore, few mechanistic experiments address how long range or *trans* interactions among PcG bound sites could occur [34], [35].

*Drosophila* Posterior Sex Combs (PSC) is likely to be central to PcG-mediated non-enzymatic effects on chromatin. PSC is part of at least three PcG complexes: Polycomb Repressive Complex 1 (PRC1) [36] and dRING associated factors (dRAF) [37], and a recently described complex including PSC, the Anaphase Promoting Complex, and Cyclin B [38]. dRAF functions as an E3 ligase for ubiquitylation of histone H2A, an activity which is essential for PcG silencing but cannot be recapitulated by PSC alone [37]. PRC1-class complexes compact chromatin [15], inhibit chromatin remodeling by ATP-dependent chromatin remodeling factors such as Swi/Snf [36], [39], and inhibit transcription [40]. PSC alone can recapitulate each of these activities [39], [40], [15]. *Psc* mutant alleles that encode for truncated proteins that lack these activities in vitro are defective in gene silencing in vivo [41]. In mES cells, deletion of PcG genes including Ring1B leads to loss of compaction at *HoxB* and *HoxD*. Expression of either wild-type Ring1B or mutant Ring1B lacking E3 ligase activity can restore compaction, suggesting compaction does not require histone ubiquitylation [33]. These data indicate that non-covalent modification of chromatin structure by PSC and related PcG proteins is likely important for PcG-mediated gene silencing. This conclusion is further supported by the finding that some PcG targets depend on PSC and Ph but not dRING for control of expression [42].

Recently, we found that PSC can form bridges between nucleosomes, holding them together after digestion of linker DNA [43]. This activity depends on both chromatin binding and self-interaction of PSC, and can occur in *trans* among mononucleosomes [43]. The ability to bridge nucleosomes could allow PSC to mediate long range or even *trans* interactions in chromatin. Here, we analyze the stoichiometry of PSC-chromatin interactions and chromatin requirements for nucleosome bridging and inhibition of nucleosome remodeling. We find that PSC compacts chromatin at a ratio of one PSC per nucleosome and further find that it can oligomerize nucleosome arrays in *trans*. Canonical chromatin folding pathways involving interactions between the tail of histone H4 and histone H2A are not required for nucleosome bridging or inhibition of chromatin remodeling. Indeed, PSC can bridge naked DNA segments, but shows a preference for nucleosomes perhaps because of its ability to interact with histone proteins. These data further define the interaction between PSC and chromatin and distinguish it from other proteins that modify chromatin conformation.

## Results

### PSC can Oligomerize Nucleosomal Arrays

**Figure 1:**
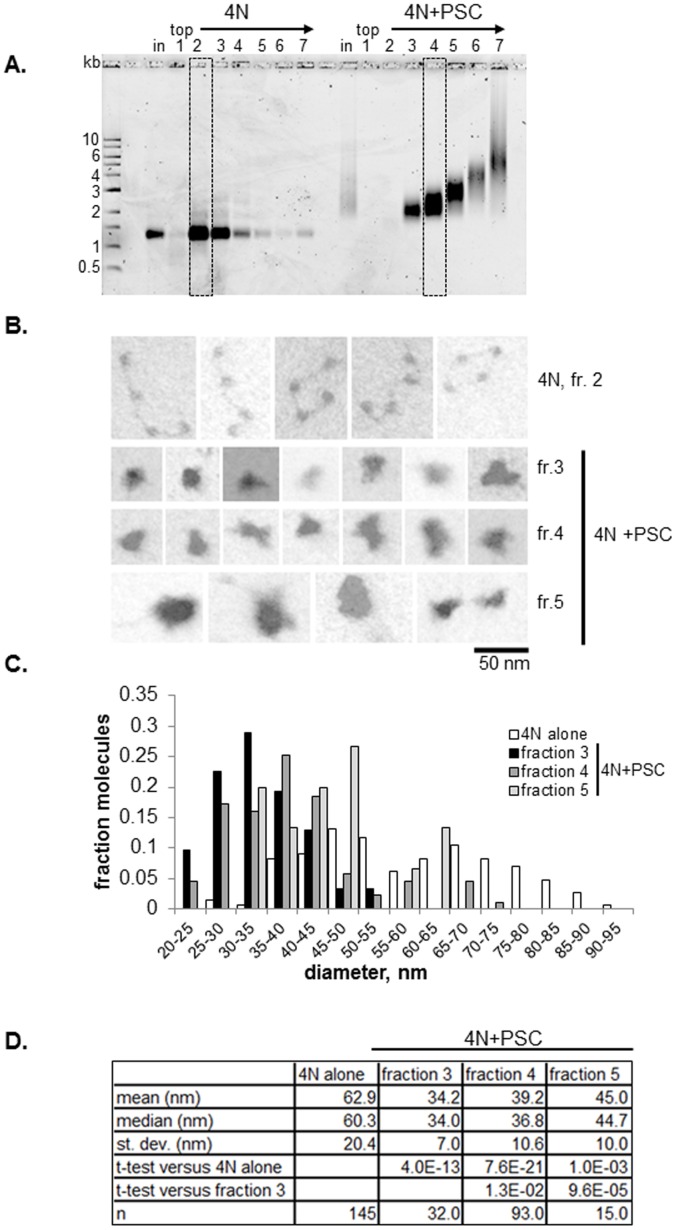
PSC compacts and oligomerizes 4-nucleosome arrays. (a) Representative glycerol gradient purification of nucleosomal templates with and without PSC for EM and STEM. Boxes indicate fractions selected for analysis. (b) Representative EM images from indicated fractions. Photographs were taken in dark field and are inverted to enhance contrast. (c) Distribution of maximum diameters of particles determined from micrographs like those in (b). Note that less than 10% of the 4N alone arrays have diameters larger than 95 nm and are not shown on the graph. (d) Summary of diameter measurements. Note that all fractions of 4N arrays incubated with PSC were significantly smaller than 4N arrays alone, and fractions 4 and 5 are different from 3. Table shows p-values for student’s t-test (unpaired, assuming equal variance in samples).

To analyze the interaction between PSC and chromatin by electron microscopy (EM) and STEM, we incubated PSC with 4 nucleosome (4N) arrays at a ratio of 0.4∶1. This concentration refers to active concentration for DNA binding; PSC preparations are typically 20–25% active assuming the protein binds DNA as a monomer. The resulting complexes were cross-linked with glutaraldehyde and fractionated by sucrose gradient sedimentation (Fig. 1a). Arrays that were not incubated with PSC sediment mainly in fractions 2 and 3. Arrays incubated with PSC were spread between fractions 3 and 7 (which includes the pellet). Analysis of these fractions on native agarose gels indicates that a series of protein-DNA complexes with progressively reduced mobility are present in the fractions. The complexes formed are discrete, suggesting they could contain different numbers of nucleosomal arrays. It is unlikely that the differences in mobility could be generated solely by binding of more PSC molecules to each array because of the low ratios of PSC to nucleosomes used, and because the amount of mass added for each PSC (169 kDa) is small relative to the mass of each array (0.9 MDa). We inspected the material in these fractions by electron microscopy. EM shows that nucleosomal arrays alone adopt an extended conformation, although 4 nucleosome circles and some more compacted forms were also observed (Fig. 1b). PSC-bound arrays form quite uniform single particles. Occasional multilobed structures were observed (bottom right image in Fig. 1b), but each lobe is of a size likely to represent an individual array. We measured the maximal diameter of arrays from three fractions (Fig. 1c) (as in [15]). PSC-chromatin complexes have significantly smaller diameters than arrays alone (Fig. 1d). Diameters increase progressively towards the bottom of the gradient, consistent with the particles having distinct compositions. Together, these results suggest the larger, single particle structures observed in lower fractions are oligomerized structures that include more than one nucleosomal array. The average diameters are much smaller than the additive diameter of two or more compacted arrays, suggesting the oligomerized structures are highly compact.

**Figure 2:**
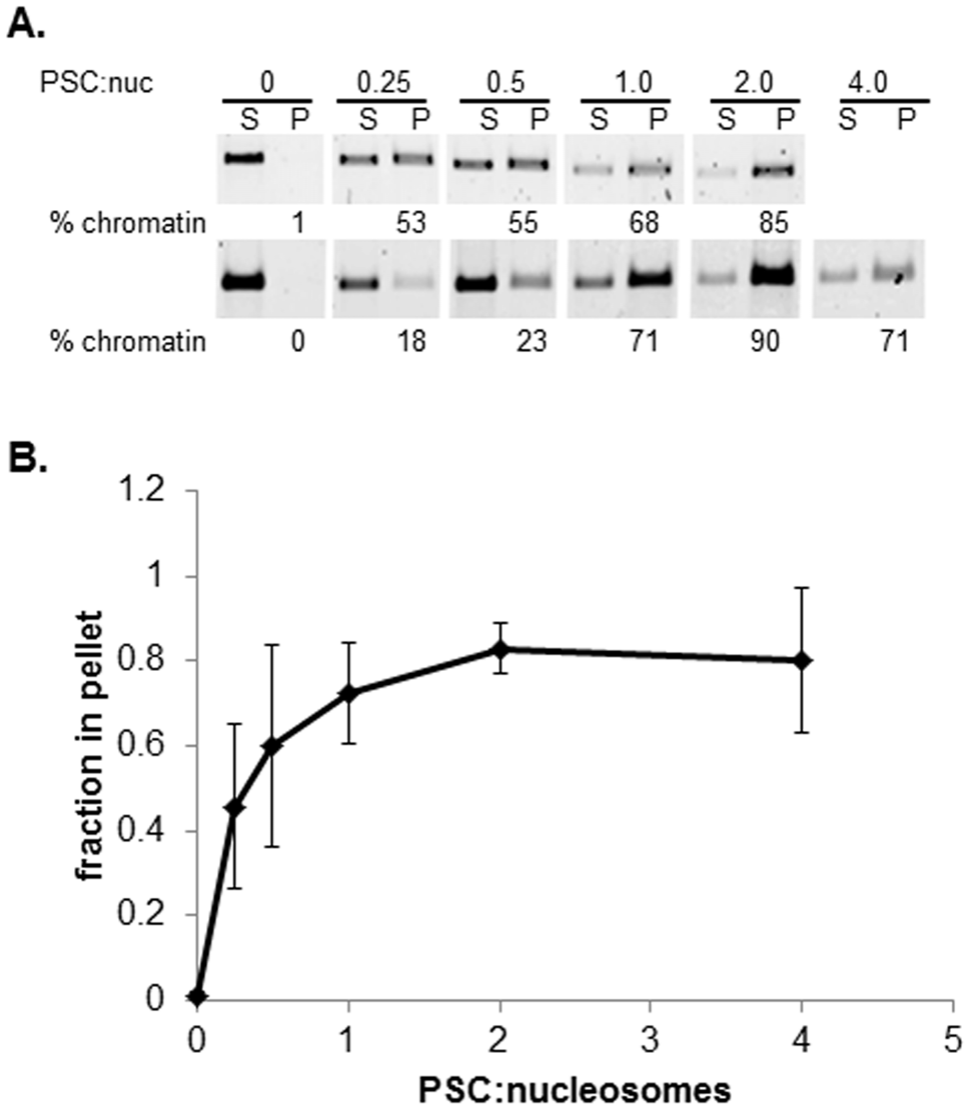
Centrifugation assay demonstrates oligomerization of 12-nucleosome arrays by PSC. (a) Nucleosomal arrays were mixed with PSC at the indicated ratios and reactions pelleted by centrifugation in a microfuge. Proteinase K digested samples were separated by agarose gel electrophoresis, stained with SYBR gold and quantified. Two different examples of the assay are shown; considerable variability was observed in this assay although the trend was constant. (b) Summary of PSC-induced oligomerization. Error bars are standard deviation in this and all other figures. n = 5.

To ask if PSC can oligomerize nucleosomal arrays using a different method, we employed a well-established centrifugation assay [44]. PSC was incubated with 12-nucleosome arrays at increasing concentrations; reactions were pelleted in a microfuge, and the fraction of the array in the pellet versus the supernatant determined. Arrays alone do not pellet under our reaction conditions, as expected. PSC also does not pellet under these conditions [15]; we centrifuged PSC at full speed prior to adding it to reactions to get rid of any large aggregates that might be present. As PSC is titrated into reactions, the fraction of arrays that pellets increases (Fig. 2). About 50% of the template pellets at a ratio of 0.25 PSCs per nucleosome, and the reaction saturates at two PSCs per nucleosome. Together, the results from different assays and with different sized nucleosomal arrays are consistent with PSC being able to oligomerize nucleosomal arrays.

### Stoichiometry of PSC and Nucleosomes in PSC-Chromatin Interaction

**Figure 3:**
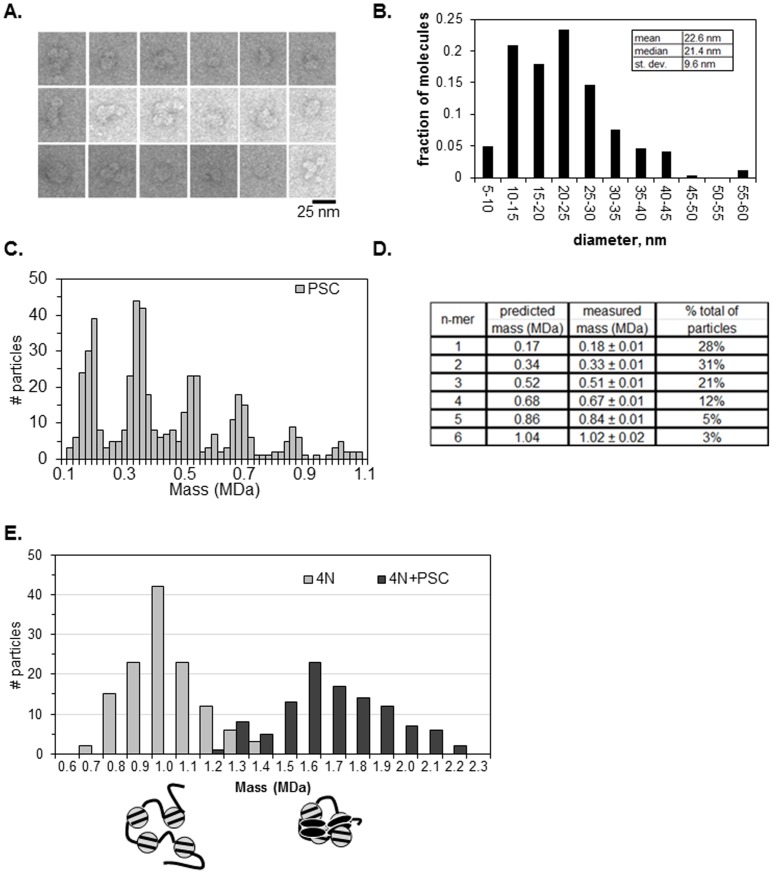
PSC compacts chromatin at a ratio of 1∶1 with nucleosomes. (a) Representative EM images of negatively stained PSC. (b) Distribution of diameters of negatively stained PSC (n = 235). (c) Mass distributions of STEM analysis of PSC alone (n = 515). (d) Summary of measured masses of PSC. (e) Mass distributions of STEM analyses. Measured masses for 4N and 4N + PSC are 0.97±0.01 MDa (n = 130) and 1.67±0.03 MDa (n = 86) respectively.

We previously determined that full inhibition of chromatin remodeling by PSC occurs at ratios of about one PSC per nucleosome, based on measurements of active concentrations [39]. However, as discussed above, we do not know what the active DNA binding form is, so that it is possible that multiple PSCs bind to single DNAs in binding assays. We therefore sought to directly measure the stoichiometry of PSC to nucleosomes in compacted chromatin that is refractory to chromatin remodeling. We used STEM to determine the ratio of PSC to nucleosomes. STEM can accurately measure particle masses using the linear relationship between electron scattering by the sample and its molecular mass [45]. We first analyzed glutaraldehyde cross-linked PSC alone by EM and by STEM. By negative staining followed by EM, particles of different sizes were observed (Fig. 3a, b). Some had complex structures but many are simple oblongs of the approximate size expected for a globular protein of 169 kDa (8–10 nm). We note that more than half of the sequence in PSC is predicted to be intrinsically disordered [46], [47], so that it is interesting that the protein has a compact rather than extended conformation.

PSC was analyzed by STEM and we observed six average measured masses in multiples of 0.17 MDa (PSC monomer predicted at 0.17 MDa) (Fig. 3c, d). These data are consistent with the protein existing in monomeric and several multimeric forms. More than 70% of the observed particles have masses consistent with multimers, consistent with previous demonstrations that PSC can self-associate [48], and with the EM data. Most of these multimers are dimers or trimers. This, along with previous data indicating that PSC forms multimers at low concentrations [48], suggests that the active DNA binding form of PSC may be a multimer.

We analyzed 4N arrays alone and with PSC (using gradient fractions similar to the boxed ones in Fig. 1a). Only fractions containing the most rapidly migrating PSC-bound species, expected to contain primarily complexes of single arrays, were analyzed (Fig. 3e). The average measured mass of 4N chromatin templates alone was 0.97±0.01 MDa, which is consistent with its predicted mass of 0.92 MDa. A single peak distribution of masses was observed for PSC +4N arrays, and the average mass of PSC-bound chromatin templates was 1.67±0.03 MDa. Thus, compacted 4N templates contain an average of 4.1 PSC molecules (expected mass = 0.92+4×0.17 = 1.6MDa). The right shoulder on the graph of PSC +4N arrays in Fig. 3e indicates that the distribution of the 4N + PSC sample tends toward higher masses. These structures could reflect more than one PSC binding to each nucleosome or binding of some nucleosomes by a PSC multimer. Nevertheless, our STEM results suggest that a minimum ratio of 1 PSC to 1 nucleosome produces a compacted species. This stoichiometry is agreement with previous estimates of the ratio of PSC to nucleosomes required to completely inhibit remodeling of a nucleosomal array [39]. This stoichiometry supports a model for nucleosome bridging in which each nucleosome is bound by PSC and these nucleosome-bound PSC interact with each other (Fig. 3e). The finding that additional PSCs can bind to arrays may explain array oligomerization since these unoccupied PSCs may function as sticky ends to capture additional nucleosomes in *trans*.

### The Acidic Patch of Histone H2A is Not Necessary for PSC-Chromatin Interactions

**Figure 4:**
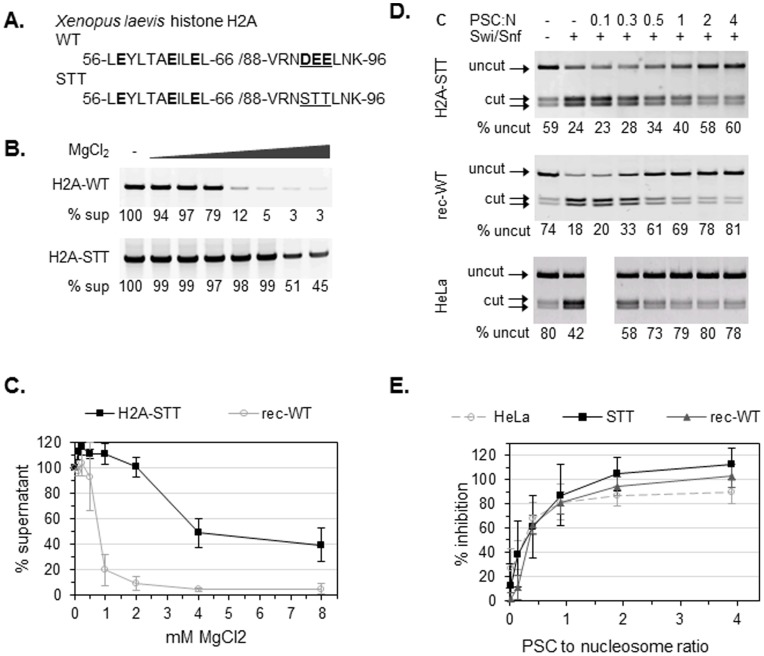
PSC does not require histone modifications or the acidic patch of H2A to inhibit chromatin remodeling. (a) Amino acid sequences for the wild-type H2A acidic patch (WT) and uncharged mutant (STT). Acidic residues are highlighted, and mutated residues are underlined. (b) MgCl_2_ dependent oligomerization of wild type and H2A-STT containing chromatin. Chromatin was incubated with the indicated concentrations of MgCl_2_ and centrifuged in a microfuge. Supernatants were electrophoresed on agarose gels and stained with SYBR gold; the % of the template remaining in the supernatant was determined by comparison with the 0 mM MgCl_2_ control. (c) Summary of chromatin oligomerization assays. (d) Restriction enzyme accessibility (REA) assays on chromatin templates with 12 nucleosomes. The chromatin template contains a unique restriction site (HhaI) that is normally occluded by nucleosomes but is exposed upon Swi/Snf-mediated chromatin remodeling. The first two lanes are negative and positive controls (with or without Swi/Snf, no PSC) demonstrating that the HhaI site becomes more accessible in the presence of Swi/Snf. (e) Summary of REA assay on chromatin templates assembled with rec-WT and H2A-STT histones. Percent inhibition is calculated as 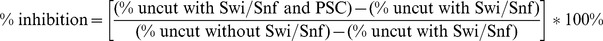.

We next investigated the mechanism PSC uses to interact with nucleosomes. Interactions among nucleosomes, particularly between the basic tails of histone H4 on one nucleosome and a cluster of acidic residues (the acidic patch) of histone H2A on another nucleosome, are believed to play important roles in chromatin folding and array oligomerization [49]–[51]. Although previous electron microscopy data showed that PSC can compact chromatin with trypsinized, tail-less histones [15], it is possible that other activities such as nucleosome bridging have different requirements than chromatin compaction. Furthermore, several nucleosome binding proteins (HMGN2, RCC1, LANA, Sir3 [52]) interact with the acidic patch of histone H2A. It is possible that PSC can interact with the H2A acidic patch to compact chromatin. PSC is a basic protein (pI = 9.2), suggesting that it could replace histone tails in nucleosome-nucleosome interactions. This mechanism would be consistent with the stoichiometry of PSC to nucleosomes, and unaffected by removal of the histone tails. To examine if nucleosome bridging and inhibition of chromatin remodeling by PSC requires the acidic patch of histone H2A, we prepared recombinant histone H2A with three key amino acids in the acidic patch (DEE) mutated to uncharged, polar residues (STT) (Fig. 4a) [51]. *Xenopus leavis* STT mutant histone H2A (H2A-STT) was prepared using standard protocols, and assembled into histone octamers with H2B, H3, and H4 [53].

Wild-type recombinant histone octamers or those assembled with H2A-STT were assembled into nucleosomal arrays on DNA containing two sets of five 5S nucleosome positioning sequences flanking a unique region (G5E4, [54]). To verify that the STT mutation disrupts chromatin folding in our hands as reported, we used the precipitation assay described above, but induced array oligomerization with MgCl_2_ instead of PSC. Previous work demonstrated that nucleosomal arrays undergo reversible oligomerization in the presence of MgCl_2_ concentrations above 1 mM [55], which depends on the H2A-H4 interaction [49], [56]. We titrated MgCl_2_ into nucleosomal arrays assembled with wild type recombinant histones (rec-WT), or H2A-STT octamers, and pelleted the arrays in an eppendorf centrifuge. Supernatants were analyzed on agarose gels, and the amount of DNA was quantified after staining with SYBR gold; each sample was compared with the control, 0 mM MgCl_2_ supernatant (Fig. 4b). We find that at least 80% of arrays assembled with rec-WT octamers are pelleted in 1 mM MgCl_2_, while only about 40% of arrays assembled with H2A-STT octamers are pelleted even at 4 or 8 mM MgCl_2_ (Fig. 4c). Thus, the chromatin assembled with H2A-STT octamers has an oligomerization defect, as reported previously [51].

We first tested whether H2A-STT interferes with the ability of PSC to inhibit nucleosome remodeling. Chromatin remodeling was monitored by restriction enzyme access (REA) to nucleosomal DNA. DNA assembled into nucleosomes is generally inaccessible [57] but can be exposed by chromatin remodeling [58]; the extent of restriction enzyme digestion is a quantitative measure of chromatin remodeling. PSC inhibits chromatin remodeling by the ATP-dependent remodeling factor hSwi/Snf [39] so that restriction enzyme digestion induced by hSwi/Snf is reduced. PSC does not substantially inhibit restriction enzyme accessibility in the absence of hSwi/Snf [39], indicating that inhibition of chromatin remodeling is specific. PSC activity on chromatin templates assembled with recombinant histones has not previously been reported, so we first compared inhibition of chromatin remodeling on templates assembled with histone octamers purified from HeLa cells or rec-WT octamers. We find that PSC inhibits remodeling of both nucleosomal arrays equally well (Fig. 4d, e), indicating that any histone modifications present on HeLa-purified octamers are not required for PSC-mediated inhibition of chromatin remodeling. We tested whether PSC can inhibit remodeling of arrays assembled with H2A-STT octamers and find that it inhibits their remodeling as efficiently as that of arrays assembled with wild-type octamers (Fig. 4d, e). Thus, neither the acidic patch of H2A nor covalent modifications present on cellular histones are required for inhibition of nucleosome remodeling by PSC.

**Figure 5:**
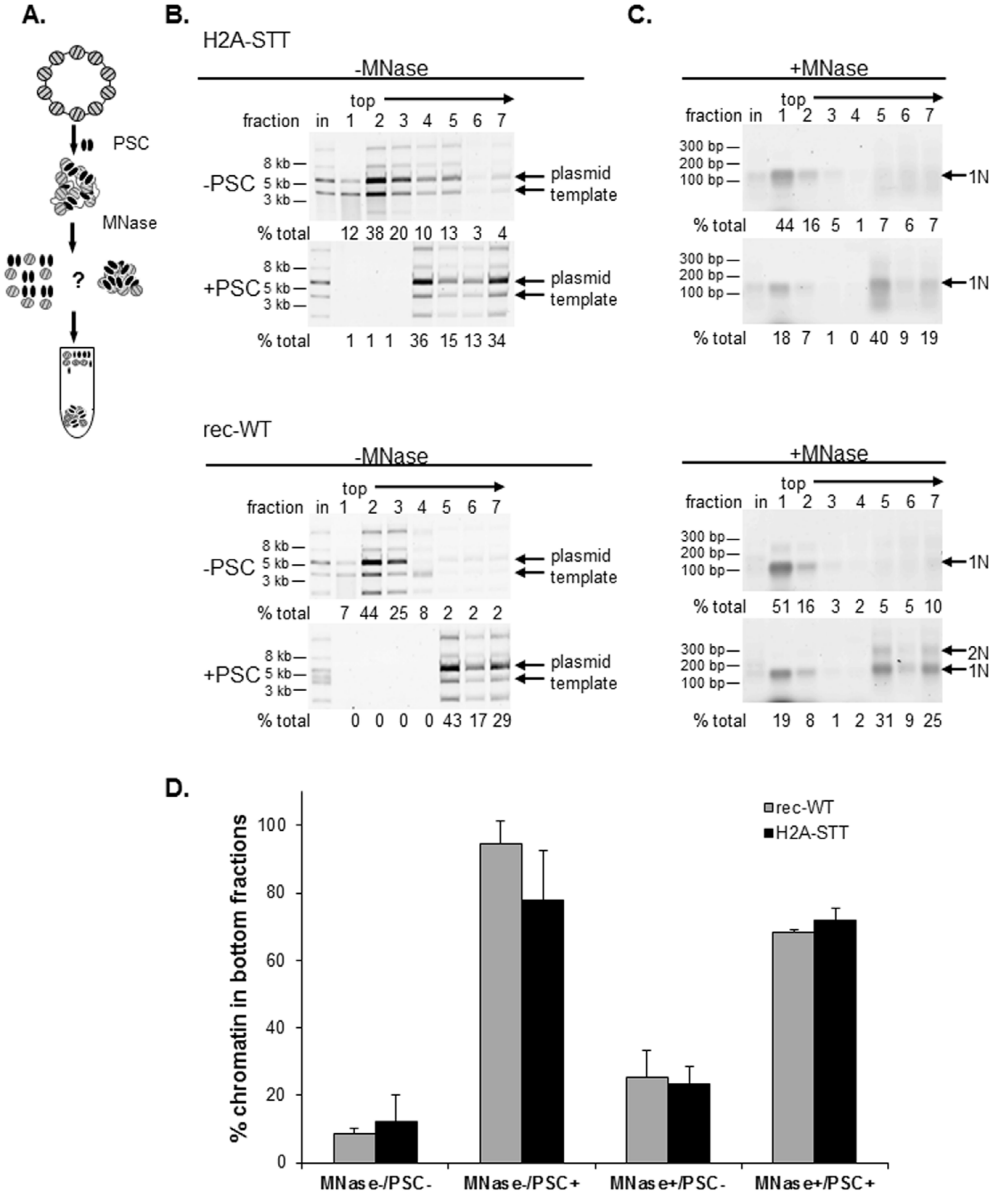
Bridging of nucleosomes by PSC does not require the acidic patch on histone H2A. (a) Schematic diagram of nucleosome bridging assay. (b) Representative control reactions for nucleosome bridging (mock MNase digested) showing that PSC binds both rec-WT and H2A-STT chromatin. Arrows point to the main plasmid forms (nicked and supercoiled); the array of minor isoforms observed here is atypical but the isoforms behave similarly. (c) Representative MNase digested nucleosome bridging reactions demonstrating that PSC bridges both rec-WT and H2A-STT nucleosomes. (d) Summary of nucleosome bridging assays on chromatin templates assembled with recombinant wild-type (rec-WT) and H2A acidic patch mutant histones (H2A-STT). Values from fractions 5–7 (bottom fractions) of sucrose gradients were summed and plotted.

We tested whether nucleosome bridging, which involves the clustering of nucleosomes, requires the acidic patch of histone H2A (Fig. 5). Nucleosome bridging is assessed on chromatinized plasmids which are incubated with buffer (control) or PSC (Fig. 5a). After PSC has bound to the chromatin, the linker DNA connecting the nucleosomes is removed by digestion with micrococcal nuclease (MNase). MNase digested reactions (or control mock-digested reactions) are sedimented through sucrose gradients which can separate bridged from free mononucleosomes, or PSC bound from unbound intact plasmids in control reactions. The nucleosome bridging assay was performed on plasmid chromatin templates with rec-WT or H2A-STT histones. Analysis of gradient fractions from reactions that were mock digested indicates that incubation of either template with PSC causes chromatin to sediment rapidly near the bottom of the gradient. Mononucleosomes generated by MNase digestion of both PSC bound templates also sediment near the bottom of the gradient. Thus, PSC is able to bridge nucleosomes from both H2A-STT and rec-WT templates with similar efficiencies (Fig. 5b–d). We conclude that neither the acidic patch nor histone modifications are required for PSC to bridge nucleosomes.

### PSC can Bridge Bare DNA Segments

**Figure 6:**
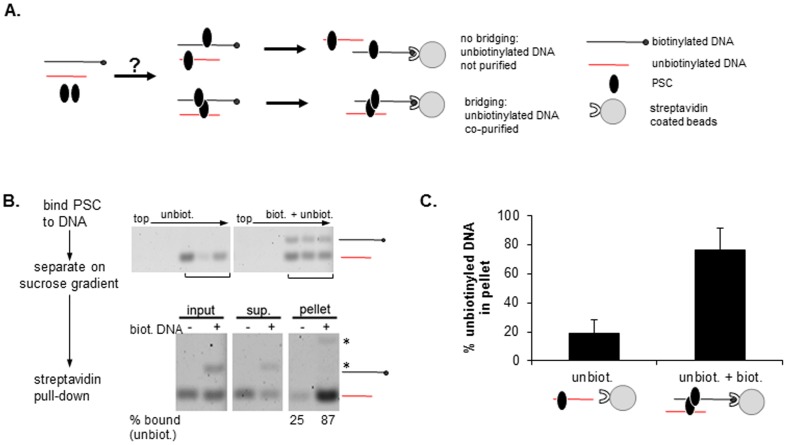
PSC can bridge bare DNA. (a) Schematic diagram of DNA bridging assay. (b) Representative analysis of bridging of naked DNA by PSC. Top panels show sucrose gradient fractions that were pooled for streptavidin pull-down. Bottom panels show streptavidin pull-down results. The per cent bound refers to how much of the unbiotinylated fragment is present in the pellet as a fraction of the total (pellet + supernatant). Asterisks indicate position of biotinylated fragment; note that in pellet fractions, biotinylated fragment is incompletely recovered by Proteinase K treatment of streptavidin coated beads, and migrates slowly likely due to bound streptavidin. (c) Summary of streptavidin pull-down experiments. Graphs show average per cent of the unbiotinylated fragment associated with streptavidin beads.

PSC binds tightly to both nucleosomes and bare DNA, suggesting its interaction with chromatin is mediated at least in part through linker DNA binding. Since our data indicate standard chromatin folding mechanisms are not required for bridging by PSC (Fig. 5b–d), we wondered if nucleosomes were required for bridging. If bridging reflects PSC-DNA binding and PSC-PSC interactions, it may not depend on nucleosomes. To ask whether PSC can bring segments of bare DNA together, PSC was incubated with either unbiotinylated DNA or a mixture of biotinylated and unbiotinylated DNAs of different sizes (Fig. 6a). Bound DNAs were isolated by sucrose gradient sedimentation (Fig. 6b). Gradient fractions were incubated with streptavidin coated beads to capture the biotinylated DNA (Fig. 6c, d). When both the biotinylated an unbiotinylated DNAs were included with PSC, both were efficiently captured by the streptavidin beads. In the absence of the biotinylated DNA, little of the unbiotinylated DNA is captured by the streptavidin coated beads even though it is still bound to PSC. Thus, PSC brings the biotinylated and unbiotinylated DNA fragments together, indicating that PSC can bridge segments of DNA as well as nucleosomes, and consistent with DNA binding playing an important role in how PSC interacts with chromatin.

### PSC Clusters Nucleosomes but also Binds DNA on Sparsely Assembled Plasmids

The observation that PSC can bridge naked DNA could indicate that it interacts with chromatin solely through DNA binding. To understand how PSC interacts with nucleosomes and DNA when both are present, we prepared 3 kb plasmid templates with a small number (1 or 2) of assembled nucleosomes and asked how PSC interacts with them by EM (Fig. S1a). Templates contain two 601 nucleosome positioning sequences, which are preferentially occupied at low ratios of histones to DNA (similar to [59]). PSC was bound at low ratios to plasmids. Templates were fixed with glutaraldehyde, rotary shadowed with platinum and visualized by EM. Titrations of PSC binding to chromatin by electrophoretic mobility shift assay (EMSA) indicate that sparsely assembled arrays readily form slowly migrating multi-template aggregates (Fig. S1b). EM was carried out with ratios of PSC that cause only a slight shift in plasmid mobility by EMSA (Fig. S1b).

**Figure 7:**
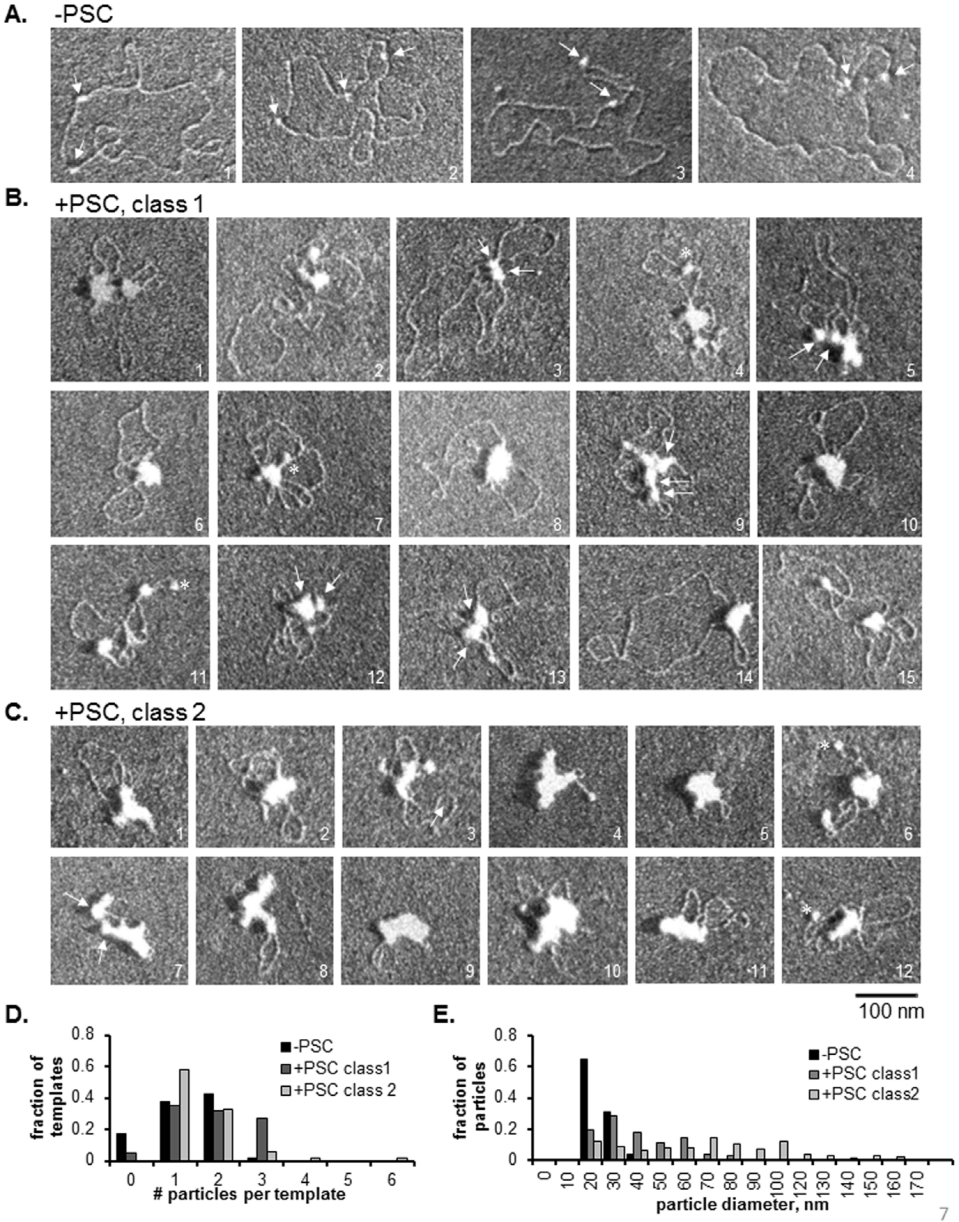
PSC clusters nucleosomes and DNA on sparsely assembled plasmids. (a) Representative EM images of plasmids with two 601 nucleosome positioning sequences assembled at low ratio of histones to DNA. Note that fully assembled plasmids would contain 17 nucleosomes and 601 sequences are separated by 385 base pairs. Plasmids were assembled in the presence of *E.coli* Topoisomerse I so that plasmids are relaxed. Arrows point to nucleosomes. Note that template 2 was the only observed example of more than 2 nucleosomes on the plasmid (3), out of the 103 molecules that were analyzed (Table 1). (b) Sparsely assembled plasmids with PSC. Class 1 molecules (see text) are more extended, and likely have fewer copies of PSC bound than Class 2 molecules (c), which are highly compacted. Arrows point to particles (likely to be PSC bound nucleosomes) that have come together and may represent the bridged configuration. Note that in some cases, more than one template may be clustered (such as molecule 3). Asterisks indicate particles that may be unbound nucleosomes (based on their size), although they could also be bound PSC. (c) Sparsely assembled plasmids with PSC with Class 2 configurations. Note that the molecules represent a series between the most extended Class 1 molecules and the most highly compacted Class 2 molecules. (d) Summary of the number of particles per template. The finding that Class 1 molecules frequently have more than 2 particles indicates that PSC must bind to naked DNA (as well as nucleosomes) on some templates. See Table 1 for summary of measurements from this and a similar experiment.

PSC formed a diverse array of structures with sparsely assembled templates (Fig. 7b, c). We classified these structures into two groups, Class 1 and Class 2, based on visual inspection of how much free DNA is visible and how large the particles on the template are. Class 1 structures likely represent templates with fewer PSC molecules bound, while Class 2 includes fully compacted structures. The simplest Class 1 structures contain one particle that is larger than the size of a nucleosome and could represent one or more PSC bound nucleosomes, and a large loop of DNA (such as molecule 14 in Fig. 7b). In some cases, small particles are observed to cluster together, which might represent simple bridging events (such as molecule 3 in Fig. 7b). Importantly, counting of the number of individual particles per template for Class 1 molecules indicates that many of these templates contain 3 particles. Templates alone contain 0, 1 or 2 particles (nucleosomes). Thus, at least some of the particles observed must represent PSC bound to DNA without a nucleosome. In contrast, most Class 2 molecules contain one or two particles, and the majority of these particles are larger than nucleosomes, and larger than the particles on Class 1 molecules (Fig. 7e, Table 1). Thus, the large particles observed in Class 2 molecules likely contain PSC bound to both nucleosomes and DNA in a compacted complex. Notably, the most compacted structures (such as molecules 4, 5, 7–11 in Fig. 7b) have very little free DNA. Together, these data indicate that PSC likely binds preferentially to nucleosomes, as observed in Class 1 molecules. PSC can also compact large regions of DNA even on templates with only one or two nucleosomes, as observed in Class 2 molecules. Because the particles on Class 2 templates are larger than those on Class 1 molecules (Fig. 7e, Table 1), the classes may represent a progression driven by binding of increasing numbers of PSC molecules.

**Table 1:** Summary of Measurements of Electron Micrographs of Chromatin with PSC.

	−PSC	+PSC class 1	+ PSC class 2	−PSC	+ PSC class 1	+ PSC class 2
**Diameter (nm, +/−SD)**	285+/−50	233+/−72	164+/−51	282+/−46	251+/−55	164+/−37
**t-test (P-value)**		8.6e-8	2.3e–26		9.3e-5	5.4e-41
**particle diameter (nm, +/− SD**	20+/−4	45+/−25	58+/−38	19+/−4	36+/−18	64+/−36
**t-test (P-value)**		3.9e–22	1.7e–21		1.5e–19	3.9e–36
**# particles (+/− SD)**	1.2+/−0.7	1.8+/−0.9	1.6+/−0.9	1.3+/−0.8	1.8+/−0.9	1.5+/−0.7
**t-test (P-value)**		3.1e–6	2.5e–3		8.9e-5	3.3e-2
**n**	100	76	43	103	62	67

Table indicates mean +/− standard deviation for three measurements from two experiments. Each template uses a plasmid containing two 601 nucleosome positioning sequences that is assembled at low ratios of histones to DNA (0.2 histone:DNA by mass). The first set template has ∼1600 bp between the 601 nucleosomes and the second has 385 bp. Diameter is the maximum diameter of each template (the diameter of the smallest circle that would completely encompass the template) [15]. The maximal diameter of each particle on each template was measured, giving rise to the “particle diameter” measurement. For –PSC samples, particles should be nucleosomes, while in +PSC samples, they could be nucleosomes, PSC bound to naked DNA, or PSC bound nucleosomes. Note that the largest diameter of the disk-shaped nucleosome is 11 nm; samples were rotary shadowed to a thickness of 3.75 nm; thus, the diameter measured is consistent with expectation (11+2×3.75 = 18.5 nm predicted size). The number of particles indicates each separate particle on a template, irrespective of size.

### PSC can Bind Free Histones

**Figure 8:**
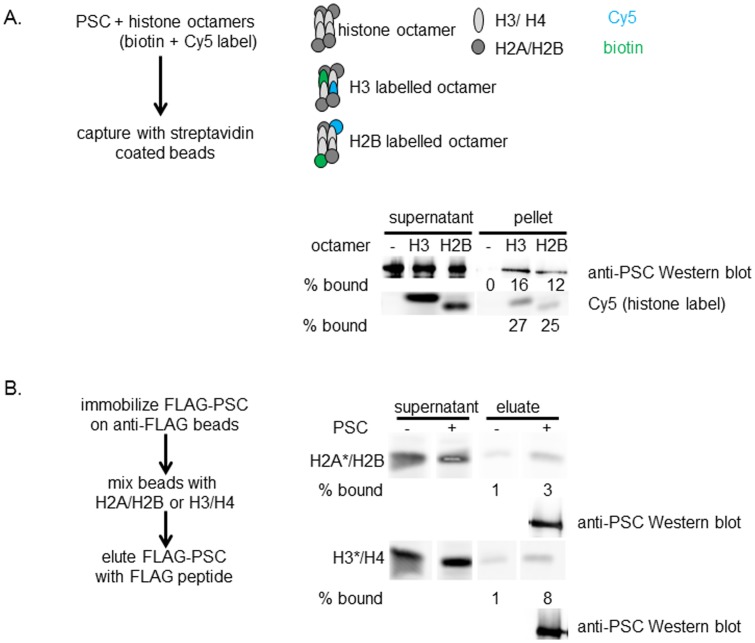
PSC can bind histone proteins. (a) Representative assay of PSC binding to histone octamers. PSC was mixed with histone octamers that contain one biotinylated and one fluorescent copy of either H3 (H3 labeled octamers) or H2B (H2B labeled octamers) (see Methods for detailed description). Mixtures were incubated with streptavidin coated beads and the amount of captured PSC determined by Western blotting. Fluorescence (Cy5) was used to monitor octamer capture. Similar results were observed in two additional assays. (b) Representative assay of PSC binding to H2A/H2B dimers or H3/H4 tetramers. Dimers and tetramers were fluorophore labeled on the indicated (asterisk) subunit. High levels of background binding to beads was observed for both dimers and tetramers, as shown, but in each of three assays, more H2A/H2B and H3/H4 were eluted from Flag beads that have immobilized PSC than control beads with no immobilized protein. PSC in the elution is detected by Western blotting.

The EM data suggest PSC has a preference for binding nucleosomes over bare DNA, and thus that PSC recognizes feature(s) of nucleosomes other than DNA. To test whether PSC might interact with the histone proteins in the nucleosome as well as the DNA, we carried out PSC binding assays with histone octamers or histone subcomplexes. We find that histone octamers bind PSC using pull-down assays (Fig. 8a). We aimed to test whether PSC binds both H2A/H2B dimers and H3/H4 tetramers; however, these assays were hindered by high non-specific binding of the histone subcomplexes to beads. By immobilizing Flag-PSC on beads, adding dimers or tetramers, washing, and then eluting Flag-PSC and bound histones (protocol from [60]), we were able to observe a low but reproducible level of specific binding to both H2A/H2B and H3/H4 (Fig. 8b). PSC binding was also observed with glutaraldehyde-mediated cross-linking (not shown). We conclude that PSC can bind to free histones although additional methods will be needed to quantitatively assess this interaction. The ability of PSC to interact with histones may contribute to its nucleosome binding activity.

## Discussion

In this paper we investigated the interaction between the essential PcG protein PSC and chromatin. The principle findings of this work are: 1) PSC can oligomerize nucleosomal arrays; 2) PSC compacts chromatin at a ratio of one PSC per nucleosome; 3) neither histone modifications nor the acidic patch of histone H2A are required for PSC to bridge nucleosomes or inhibit chromatin remodeling; 4) PSC can bridge naked DNA; 5) PSC can interact with histones.

**Figure 9:**
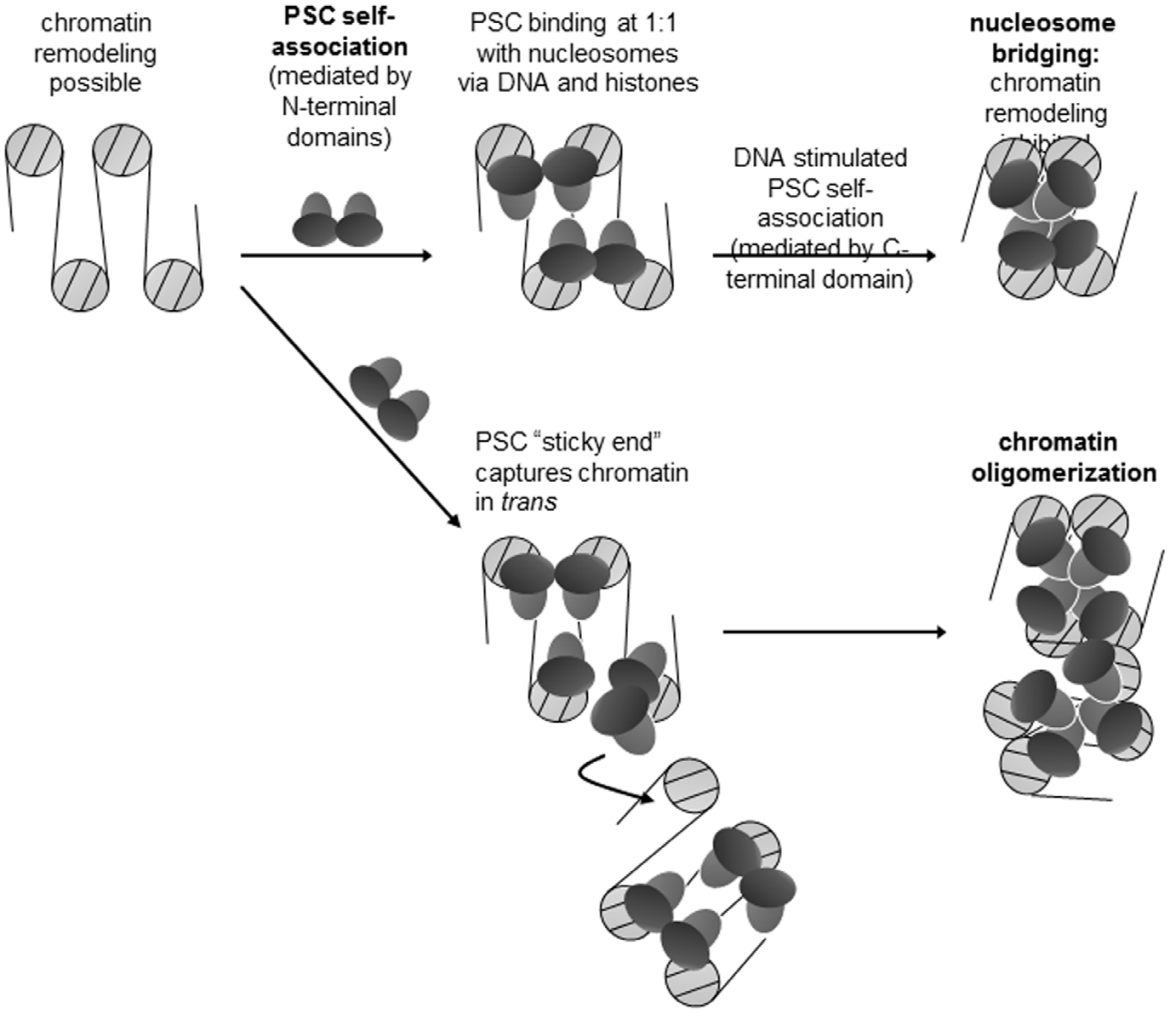
Model for nucleosome bridging and chromatin oligomerization by PSC. See discussion for details.

Taken together, our results and previous findings support the following mechanism (Fig. 9). PSC binds to chromatin using interactions with DNA and one or more histones, at a stoichiometry of one PSC per nucleosome. Interactions between PSC molecules then bring PSC-bound nucleosomes together. Because PSC alone can form multimers at low concentrations [48], “extra” PSC can associate with nucleosomal arrays through interactions with nucleosome-bound PSC molecules; these “extra” PSCs may function as sticky ends to promote oligomerization of arrays in *trans*. Notably, the ratios of PSC to nucleosomes required for compaction and oligomerization are very similar. This suggests that oligomers may form through the same mechanism as compacted chromatin–with nucleosome bridges forming in *trans* (as we have shown they do with mononucleosome templates [43]), but not requiring an extra “layer” of PcG protein. PSC can also bring segments of naked DNA together and, on templates with few nucleosomes, PSC can gather DNA together with nucleosomes into highly compacted structures, and form template oligomers. This indicates that a gap in nucleosomes, as occurs at regions of high nucleosome turnover at PREs [17], [61], [62], should not impede compaction by PSC. In these experiments, none of the physiological targeting mechanisms for PSC are in play. Instead the proteins simply bind tightly but non-specifically to chromatin so that it is likely that arrays are first bridged and then compacted after all of the nucleosomes are “filled”. In the more complex physiological situation such as occurs in vivo, it is possible that nucleosome bridging activity is restricted or targeted in a way that favors interactions among distal PREs rather than local spreading of an altered configuration.

We previously analyzed interactions between the PRC1 core complex (PCC), which contains PSC and two other PcG proteins, and a 4N array by STEM. We found that one complex was associated with single, compacted 4 N arrays. This suggests that the stoichiometry of PSC alone versus in a complex may be different, which is also consistent with the small difference in concentration of PCC versus PSC required to inhibit chromatin remodeling [39]. PSC can self-interact through both its N and C-terminal regions [43]. The N-terminal self-interaction activity likely involves the conserved RING finger of PSC. This domain is also involved in forming PCC, and it is unlikely to self-interact in the context of PCC. The C-terminal self-interaction activity is housed in the large unstructured region of PSC. This activity is stimulated by DNA [43]. The use of only PSC’s C-terminal self-interaction activity by PCC may account for differences in its behavior as compared with PSC. The protein MeCP2, which also compacts chromatin [63], was also analyzed on 4N arrays by EM and STEM. MeCP2, at ratios of 2 copies per nucleosome, promoted formation of folded “bow-tie” like structures of single 4N arrays. These structures likely represent a zig-zag conformation of the arrays, similar to the conformation captured in a crystal structure of a tetranucleosome [3]. MeCP2 can compete with linker histone for binding to nucleosomes, protects linker DNA, and may compact chromatin by interacting with linker DNA to promote chromatin folding [3]. The bow-tie conformation was not apparent in 4N arrays incubated with PSC, and PSC does not create a chromatosome stop at 165 base pairs the way linker histone does (e. g. Fig. 5) although linker DNA enhances PSC binding to nucleosomes [48].

Two other chromatin proteins that can compact chromatin use distinct mechanisms from PSC. The *S. cerevisiae* protein Sir3p binds both DNA and chromatin, and Sir3p can multimerize. It forms complex compacted structures with chromatin [2], [64]. Sir3p also forms unique structures on DNA that are not formed by PSC [2], [64]. Recently, the structure of the BAH domain of Sir3p bound to a nucleosome was solved [65]. This revealed that this domain binds to the face of the nucleosome, including contacts with the acidic patch of histone H2A [65]. This binding may mediate chromatin compaction through face-to-face interactions of Sir3p bound nucleosomes. The human L3MBTL1 protein, which contains MBT domains that recognize methylated histone tails, can also compact chromatin [59]. Again the mechanism is distinct from that of PSC in that single copies of L3MBTL1 are believed to interact with two nucleosomes through binding of the MBT domains to histone tails [59]. This mechanism effectively locks nucleosomes together, compacting the chromatin, and is also unique in being dependent on histone methylation. These examples emphasize how chromatin compaction does not arise through a single mechanism. Instead, there are likely many different chromatin architectures that are “compacted”, each of which may serve distinct regulatory functions.

In *Drosophila* imaginal discs, PcG proteins are bound at target genes in both the on and off states, suggesting that PcG repression of transcription is regulated at a step after recruitment [61]. We hypothesize that regulation of nucleosome bridging or *trans* interactions could be part of this regulation. For example, the *O*-GlcNAc transferase (OGT) enzyme, which adds the *N*-acetylglucosamime sugar group to serine residues, was recently identified as an essential Polycomb protein encoded by the *super sex combs* (*sxc*) gene [66], [67]. Mutations in *sxc* disrupt PcG-mediated silencing but not PcG protein binding [66], indicating that post-translational modification of PcG proteins including PSC by this enzyme, or other enzymes, may regulate their activities on chromatin. It is also possible that histone modifications, such as the H3K27me3 that is associated with PcG silencing and recognized by a subunit of PRC1, can modulate chromatin architecture induced by PSC. Defining how proteins like PSC alter chromatin architecture sets the stage to investigate regulation of these mechanisms.

## Methods

### Protein Expression and Purification

FLAG epitope-tagged PSC and truncations were expressed in Sf9 cells using the Bac-to-Bac Baculovirus Expression System (Invitrogen) and purified as described [39]. HeLa histone octamers were purified as described [68]. Recombinant wild-type and mutant *Xenopus laevis* histones were purified individually and refolded into octamers as described [69]. H2A acidic patch triple mutant (D91S, E92T, E93T) was generated by QuikChange XL site-directed mutagenesis (Stratagene). Fluorescently labeled histones were prepared from recombinant octamers that contain an engineered cysteine (H3–33, H2A119, H2B 120). Swi/Snf was prepared as described [70].

### Filter Binding

Double filter analysis of DNA binding [71] was carried out with ^32^P-labeled 157-bp TPT fragment as described [39]. Filters were quantified on a Typhoon Trio Variable Mode Imager (GE Healthcare) and by ImageQuant TL (GE Healthcare). Active fractions of PSC were determined using DNA in excess of protein and were typically close to 20%. All stated concentrations refer to active concentrations.

### Chromatin Assembly

Plasmid pG5E4-SVO [72] and linear DNA templates for 12 nucleosome templates [54], [15] were described. The four-nucleosome template composed of 4 copies of the 601 nucleosome positioning sequence used for STEM analysis was a generous gift from C. Woodcock (University of Massachusetts, Amherst) [3]. Nucleosomal arrays were assembled with purified HeLa or recombinant histone octamers by gradient salt dialysis as described [73]. Mononucleosomes were assembled as described [74].

### Electron Microscopy (EM) and Scanning Transmission EM (STEM)

Samples for EM visualization were prepared as described [15]with an additional purification through step glycerol gradients (10%/20%/30%) prepared in HEN buffer (10 mM HEPES pH 7.9, 0.25 mM EDTA, 2.5 mM NaCl) at 30,000 rpm for 90 min at 4°C in rotor TLS-55. Selected fractions from gradients were applied to glow-discharged, carbon-coated grids, and stained with uranyl acetate and rinsed with water to achieve a positive stain as described [75].

STEM analysis was carried out at the Brookhaven National Laboratory (BNL) as described [15] on the same materials used for EM. Scattering data were collected at 1.0 or 0.5 nm^2^/pixel, and particle masses were measured using tobacco mosaic virus (TMV) as an internal standard [45] by PCMass29 (BNL). STEM measurements were carried out on a single preparation of PSC and chromatin; however, the STEM samples are representative of multiple samples that were analyzed by EM.

### Restriction Enzyme Accessibility (REA) Assay

Experiments were performed as described [58], [39] with affinity purified hSwi/Snf and the restriction enzyme HhaI. Reaction conditions were as follows: 12 mM Hepes, pH 7.9, 12% glycerol, 0.24 mM EDTA, 60 mM KCl, 4 mM MgCl_2_, 2 mM ATP, 1mM DTT, and 0.015-0.03% NP40. At the end of the remodeling reaction, proteins were removed by incubation with DSB (25% glycerol, 50 mM Tris pH 8.0, 100 mM EDTA, 1% SDS, bromophenol blue, and xylene cyanol) and 30 µg proteinase K (PK) (Bioneer) for 60 min at 50°C. DNA was resolved in agarose gels and visualized with SYBR gold stain (Invitrogen) on a Typhoon Imager. All experiments were performed at least three times with two different preparations of proteins and chromatin.

### Nucleosome Bridging Assay

Chromatinized plasmids containing 80 nM nucleosomes were incubated with PSC at 0.5–1.0 molar ratio in standard reaction conditions (identical to REA conditions but without ATP and with 2 mM MgCl2). Linker DNA was digested with the addition of 1U MNase (USB), 10 mM CaCl_2_, and 0.5 µg of a 29-bp DNA competitor at 30°C for 5–10 min. Reactions were stopped with 15 mM EDTA and separated in step sucrose gradients (20%/40%/80%) prepared in BC buffer (20 mM HEPES pH7.9, 0.2 mM EDTA) containing 0.025% NP40 and 60 or 150 mM KCl at 40,000 rpm for 50 min at 4°C in rotor TLS-55 (Beckmann) in an Optima TL tabletop ultracentrifuge. Fractions were collected, and proteins were removed by PK digest as above. DNA was resolved in agarose gels and visualized with SYBR gold stain (Invitrogen) on a Typhoon Imager.

For DNA bridging, PSC was incubated with DNA fragments (35 nM PSC, 40 nM DNA fragment), and binding reactions centrifuged through step gradients as for nucleosome bridging. Selected gradient fractions (determine by running aliquots of fractions on DNA gels) were normalized for sucrose concentration and incubated with Dynabeads M-280 Streptavidin (Invitrogen) for 2 hours at 4°C with 0.5 mg/ml BSA. Beads were washed twice with BC buffer containing 20% glycerol, 0.025% NP40, 0.5 mg/ml BSA and 60 mM KCl, and proteins were removed with DSB and proteinase K as for the nucleosome bridging assay; gels were stained with SYBR gold to visualize DNA.

### Histone Binding

Histone octamers were prepared using standard methods [69]except that one subunit (either H2B-122 or H3–33) was labeled with a fluorophore or biotin through a cysteine substitution [76]. Octamers were prepared with equal amounts of fluorophore and biotin labeled subunit. Thus, for example, most H3 labelled octamers contain one H3-Cy5 and one H3-biotin. PSC was mixed with an excess of histone octamers for 30 minutes at room temperature, and then added to BSA blocked streptavidin coated beads for 30 minutes at room temperature with rotation. Histone octamers are prepared in 2M NaCl so that binding assays were carried out at 200 mM NaCl and 30 mM KCl. Beads were washed three times in binding buffer with 60 mM KCl. Beads and supernatants were boiled and loaded onto SDS-PAGE gels. Anti-PSC [72] was used to assess how much PSC associates with beads in the presence or absence of the biotinylated octamers. To assess binding to H2A/H2B or H3/H4, we used the protocol described by Belotserkovskaya et al. [60]. H2A/H2B with uniformly Cy3 labelled H2A (at position 119) and H3/H4 with uniformly Cy5 labelled H3 (position 33) were prepared similarly to octamers and purified by gel filtration. Flag-PSC was immobilized on Flag beads, mixed with an excess of histones, washed, and PSC eluted with 0.4 mg/ml of Flag peptide. Eluates were electrophoresed on SDS-PAGE gels and scanned to detect the fluorescent labels on H2A or H3 on a Typhoon Imager. Western blotting was used to confirm capture and elution of Flag-PSC.

## Supporting Information

Figure S1 **Characterization of sparsely assembled templates used for** **Figure 7** **.** a) Restriction enzyme digest to liberate 601 fragments followed by native gel electrophoresis demonstrates the degree of nucleosome assembly on 601 sequences. Schematic diagrams indicate positions of 601 nucleosome positioning sequences (grey circles); restriction sites that flank the 601 sequences are indicated with red slashes. Note that the plasmids contain additional sites for these enzymes giving rise to the complex digest pattern. C = chromatin; D = DNA. Black box indicates the band containing the 601 sequence; red box indicates the 601 band with an assembled nucleosome (note this band is not present in the bare DNA lanes). The additional extra band at about 1 kb in the chromatin lanes likely represents a partially digested fragment containing a 601 sequence, although we cannot rule out the possibility that it is a different fragment of the plasmid that preferentially assembles a nucleosome. b) EMSA of PSC binding to sparsely assembled templates. Glutaraldehyde cross-linked samples were separated on a 0.8% agarose, 0.5X TBE gel and stained with SYBR gold. Reactions used for EM are indicated with arrows. Note that at the highest concentration of PSC, the large template aggregates that were formed do not enter the gel.
(TIF)
