# Supplementary figures and images for: Chromatin Modification by PSC Occurs at One PSC per Nucleosome and Does Not Require the Acidic Patch of Histone H2A
Source: PLoS One. 2012 Oct 11;7(10):e47162. doi: 10.1371/journal.pone.0047162 (PMC3469540; doi:10.1371/journal.pone.0047162)

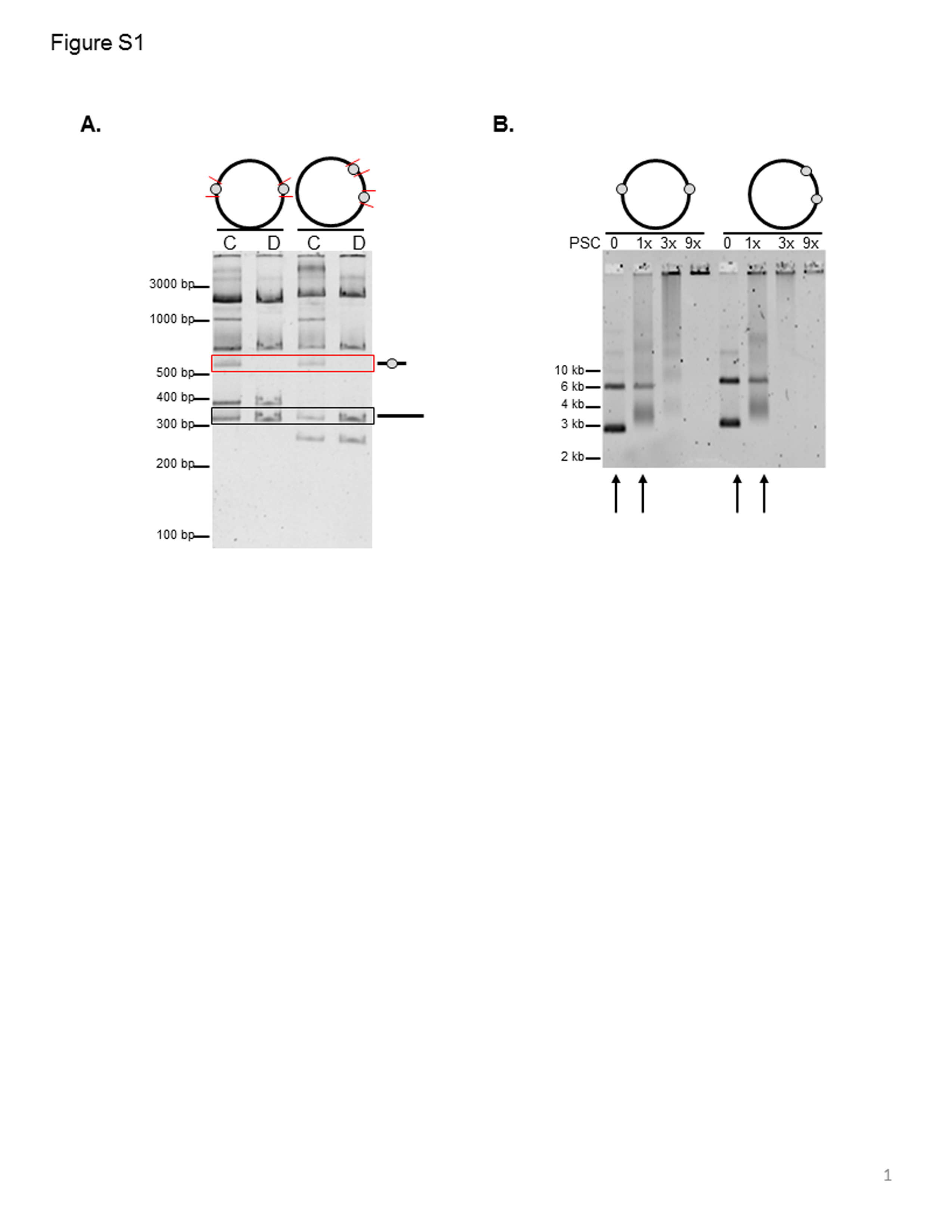

Supplement: Figure S1 — Characterization of sparsely assembled templates used for Figure 7 . a) Restriction enzyme digest to liberate 601 fragments followed by native gel electrophoresis demonstrates the degree of nucleosome assembly on 601 sequences. Schematic diagrams indicate positions of 601 nucleosome positioning sequences (grey circles); restriction sites that flank the 601 sequences are indicated with red slashes. Note that the plasmids contain additional sites for these enzymes giving rise to the complex digest pattern. C = chromatin; D = DNA. Black box indicates the band containing the 601 sequence; red box indicates the 601 band with an assembled nucleosome (note this band is not present in the bare DNA lanes). The additional extra band at about 1 kb in the chromatin lanes likely represents a partially digested fragment containing a 601 sequence, although we cannot rule out the possibility that it is a different fragment of the plasmid that preferentially assembles a nucleosome. b) EMSA of PSC binding to sparsely assembled templates. Glutaraldehyde cross-linked samples were separated on a 0.8% agarose, 0.5X TBE gel and stained with SYBR gold. Reactions used for EM are indicated with arrows. Note that at the highest concentration of PSC, the large template aggregates that were formed do not enter the gel. (TIF) [file pone.0047162.s001.tif]
